# Circulating *PIK3CA* mutation detection at diagnosis in non-metastatic inflammatory breast cancer patients

**DOI:** 10.1038/s41598-021-02643-y

**Published:** 2021-12-15

**Authors:** Violette Allouchery, Anne Perdrix, Céline Calbrix, Anca Berghian, Justine Lequesne, Maxime Fontanilles, Marianne Leheurteur, Pascaline Etancelin, Nasrin Sarafan-Vasseur, Frédéric Di Fiore, Florian Clatot

**Affiliations:** 1 Department of Medical Oncology, Centre Henri Becquerel, 1 Rue d’Amiens, 76038 Rouen Cedex 1, France; 2 IRON Group, Inserm U1245, UNIROUEN, Rouen University Hospital, Normandy Centre for Genomic and Personalized Medicine, Normandie Université, Rouen, France; 3 Department of Bio-Pathology, Centre Henri Becquerel, Rouen, France; 4 Department of Biostatistics, Rouen University Hospital, Rouen, France; 5 Department of Gastroenterology, Rouen University Hospital, Rouen, France

**Keywords:** Cancer, Molecular biology, Oncology

## Abstract

Inflammatory breast cancer (IBC) is an aggressive BC subtype with poor outcomes. A targetable somatic *PIK3CA* mutation is reported in 30% of IBC, allowing for treatment by PI3Kα-specific inhibitors, such as alpelisib. The aim of this study was to evaluate the detection rate of circulating *PIK3CA* mutation in locally-advanced IBC (LAIBC) patients harbouring a *PIK3CA* mutation on initial biopsy. This monocentric retrospective study was based on available stored plasma samples and tumour biopsies at diagnosis from all LAIBC patients treated with neo-adjuvant chemotherapy (NCT) between 2008 and 2018 at the Centre Henri Becquerel. *PIK3CA* mutations (E542K, E545K, H1047R/L) were assessed by droplet digital PCR (ddPCR) in plasma samples and tumoral tissue at diagnosis. A total of 55 patients were included. Overall, 14/55 patients (25%) had a *PIK3CA* mutation identified on baseline biopsy (H1047R = 8; H1047L = 3; E545K = 2; E542K = 1). Among them, 11 (79%) patients had enough DNA for circulating DNA analyses, and corresponding circulating *PIK3CA* mutations were found in 6/11 (55%). Among the 41 patients without *PIK3CA* mutations on biopsy, 32 (78%) had enough DNA for circulating DNA analysis, and no circulating *PIK3CA* mutation was identified. Our results revealed no prognostic or predictive value of *PIK3CA* mutations at the diagnosis of non-metastatic IBC but highlighted the prognostic value of the cfDNA rate at diagnosis. Our study showed that a corresponding circulating *PIK3CA* mutation was identified in 55% of LAIBC patients with *PIK3CA*-mutated tumours, while no circulating mutation was found among patients with *PI3KCA* wild-type tumours.

## Introduction

Inflammatory breast cancer (IBC) is a rare form of breast cancer that accounts for approximately only 2% to 4% of all cases^1–3^ and contributes to 10% of breast cancer-caused mortality^4^. IBC is characterized by an early age at diagnosis, aggressiveness and poor survival^5,6^. Data on IBC risk factors are limited, but there is a higher incidence in young African-American women, and a high body mass index (BMI) is more frequently associated with IBC than with non-inflammatory breast cancer^7^. Originally described by Sir Charles Bell in 1814^8^, the diagnosis of IBC is commonly based on clinical criteria, described by the American Joint Committee of Cancer (AJCC) as rapid onset of breast skin erythema with oedema (known as “peau d’orange”) and considered T4d stage according to TNM classification. IBC patients also have more frequent lymph node involvement, and 30% are metastatic at diagnosis^9,10^.

Treatment is multimodal, including neoadjuvant chemotherapy (NCT) followed by mammectomy with axillary dissection if a tumour-free resection margin is expected and locoregional radiotherapy^11^. Until now, the median overall survival (OS) of IBC patients has remained poor, with a median OS of 43 months in the entire population^12^. Although the presence of a pathological complete response (pCR) after NCT is considered a significant prognostic factor in all biological subtypes of IBC^13^, there is no consensus predictive marker of pCR. For the past 20 years, several studies have tried to provide a molecular description of IBC^14^ but were relatively limited by the rarity of this entity and the small sample size. Compared to non-inflammatory BC, IBC is generally characterized by important genomic instability and a lower frequency of luminal A subtypes^15^. However, IBCs do not share a specific pattern of molecular alteration^16^. The most frequent somatic mutations are those located on the *TP53* and *PIK3CA* genes, which are observed in approximately 75% and 40% of cases and have a higher prevalence than within non-inflammatory breast cancer^17^.

*PIK3CA* activating mutation induces hyperactivation of the alpha isoform (p110alpha) of phosphatidylinositol-3-kinase (PI3K) and activates the PI3K/AKT/mTOR pathway, which is the most frequently activated pathway in breast cancer and one of the most important mechanisms in endocrine therapy resistance^18^. *PIK3CA* mutations are found in 22 to 30% of breast cancers^19^ and in 40% of hormone receptor-positive (HR +) HER2− tumours^20,21^. More than 90% of these mutations are restricted to two hotspots: E542K or E545K in exon 9 and H1047R or H1047L in exon 20^22^, which are easily identified by sensitive methods such as digital PCR. In the era of liquid biopsy, a high concordance between tumour tissue and circulating tumoral DNA (ctDNA) mutation status^23,24^ has been reported. Moreover, while the prognostic value of *PIK3CA* mutations remains controversial^21,25^, their predictive value as a marker of response to PI3K pathway inhibitors is now established^26,27^. In particular, the PI3Kα-specific inhibitor alpelisib has recently shown manageable toxicity and good clinical activity in *PIK3CA*-mutated BC. Moreover, patients with circulating *PIK3CA* mutations rather than biopsy-based *PIK3CA* mutations have a better predictive value for response to PI3K inhibitors^28,29^. In this context, the aim of this study was to investigate the association and the clinical impact of *PI3KCA* mutational status in paired tumour and plasma samples at diagnosis in patients with locally advanced IBC (LAIBC) undergoing NCT.

## Methods

### Patients

We retrospectively screened all patients with LAIBC undergoing NCT at the Centre Henri Becquerel from 2008 to 2018. IBC was defined by clinical stage T4d, and pathological evidence of tumour emboli in the dermal lymphatics was not mandatory. Only patients with available tumour tissue from diagnostic biopsies and corresponding blood sample collection were included in the analysis dataset. Tumour biopsies and corresponding plasma samples at diagnosis were analysed for *PIK3CA* mutations using ddPCR. *PIK3CA* mutation status was also analysed in surgical resections and plasma samples after neoadjuvant treatment when available in patients with *PIK3CA-*mutated BC at diagnosis. The last update for survival follow-up was July 2020.

This study was conducted in accordance with French laws regarding retrospective studies. All patients received a non-opposition form, and the study was authorized by our local institutional review board (IRB) (Centre Henri Becquerel, No. 1913B).

### DNA extraction in formalin-fixed, paraffin-embedded (FFPE) and plasma samples

DNA of FFPE breast tumour biopsies was extracted with the Maxwell 16FFPE Plus LEV DNA Purification Kit (Promega, Madison, Wisconsin, USA) using two cuts of 2 µM.

Blood samples were remnants of blood analyses performed during IBC patient treatment. Blood samples were collected in heparinized or EDTA tubes and processed within two hours after collection with one centrifugation at 2000 *g* ^10min^ at 4 °C before storage at −20 °C. cDNA was isolated using the QIAamp Circulating Nucleic Acid Kit (Qiagen, Hilden, Germany). Double-stranded DNA quantification was performed by the fluorometric method using a Qubit ds DNA HS Assay Kit (Thermo Fisher Scientific, Waltham, MA, USA). DNA was pre-amplified as previously described^30,31^.

### ddPCR

Analyses for *PIK3CA* mutation detection were performed blind to the clinical data. ddPCR from the Stilla system (Stilla Technologies, Villejuif, France) was used for *PIK3CA* mutation detection in the plasma and FFPE samples. We used a Bio-Rad (Hercules, CA, USA) ddPCR assay for the four mutations, E542K (dHsaMDV2010073), E545K (dHsaMDV2010123), H1047R (dHsaMDV2010077), and H1047L (dHsaMDV2010123).The results were analysed using CrystalMiner software (Stilla Technologies, Villejuif, France) which enables a visualization of each chamber (visualization of the droplets appearing as empty, wild-type positive or mutant positive) and provides a count of the generated droplets, the positive droplets for wild-type signal and the positive droplets for mutant signal. The variant allele fraction (VAF) was defined as the proportion of mutant DNA copies compared with wild-type (WT) DNA copies obtained by ddPCR. To validate the run, we verified 2 criteria: a minimum of 15,000 total droplets generated, and a minimum of 200copies/µL (wild type copies + mutant copies) obtained. In contributive runs according to these 2 previous criteria, we confronted the number of mutant positive droplets to the limit of detection (LOD) value: the sample was considered as positive if the number of positive droplets was larger than the LOD. Each sample was tested in duplicated. For every duplicate, the same qualitative conclusion (mutated or not mutated) was obtained. The VAF mentioned in Table 2 represents the mean of the 2 duplicates.

### Statistics

The primary endpoint was the association between *PIK3CA* mutation status at diagnosis between tumour tissue and corresponding plasma. The key secondary endpoints were to evaluate the association between *PI3KCA* mutational status and IBC molecular subtype, pathologic response and disease-free survival (DFS) and overall survival (OS). The impact of the total circulating DNA level at diagnosis on the pCR rate, DFS and OS was also analysed as well as the association between pCR and survival. pCR was considered in our study as the absence of invasive disease after mastectomy and lymphadenectomy (ypT0/is, N0), according to the Residual Cancer Burden calculator of the MD Anderson Center. DFS and OS were defined as the time from diagnosis to relapse, death, or death only, respectively. Patients were defined as refractory in the absence of a response to neoadjuvant chemotherapy, including clinically progressive disease and stable disease.

The chi-square test was used for the comparison of patient characteristics according to their mutational status at diagnosis. The Kaplan–Meier method was used to estimate the DFS and OS endpoints. The log-rank test was used to compare survival curves according to the observed determinants. P-values < 0.05 were considered significant. All reported P-values are two-sided, and confidence intervals (CIs) are at the 95% level. Statistical analyses were performed using R statistical software (version 4.0.2).

### Ethics approval and consent to participate

Informed patient consent was obtained by sending a non-objection form. The study was approved by the Institutional Review Board of the Henri Becquerel Center (register order 1913B).

## Results

### Patient characteristics

**Figure 1:**
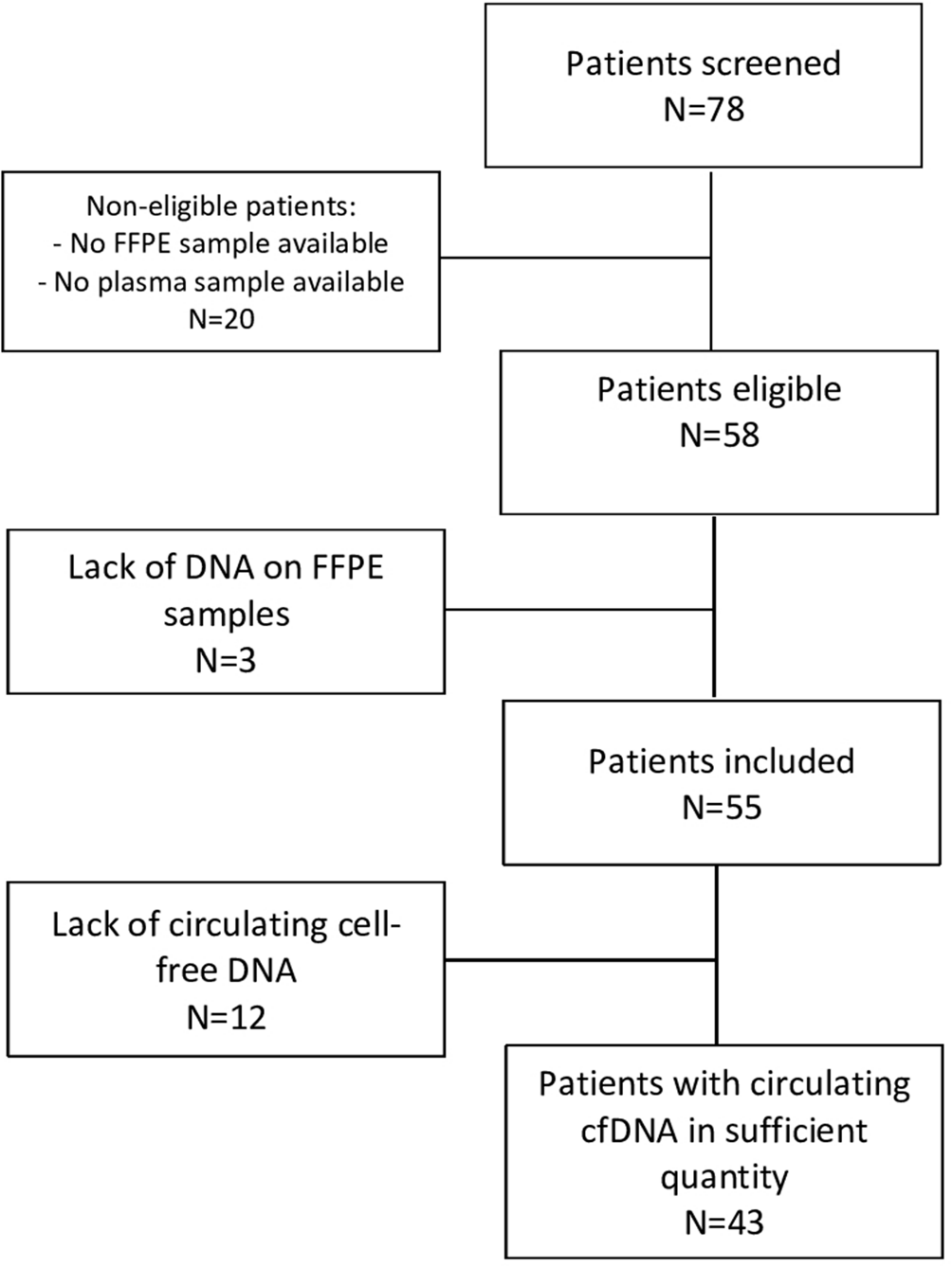
CONSORT diagram. Among the 78 patients screened, 20 were non-eligible because of non-available FFPE samples or plasma samples. Among them, 3 had a lack of DNA on FFPE samples and 55 patients were included. Finally, there was a lack of circulating cell-free DNA for 12 patients with 43 patients with circulating cfDNA in sufficient quantity.

A total of 55 LAIBC patients were considered for this study, and 43/55 (78%) had sufficient quality samples for tumour and circulating DNA analyses, as illustrated in the CONSORT diagram of Fig. 1. The main characteristics of the population are summarized in Table 1. The median age was 55 years (range 33–87), with 43.6% premenopausal patients. Most LAIBC patients were obese with a median BMI of 30.6 kg/m^2^ and had an aggressive profile with high tumour grade, lymph node invasion and a higher rate of HR-negative tumours.

**Table 1:** Characteristics.

		Total N = 55	FFPE *PIK3CA* mutated patients N = 14	FFPE *PIK3CA* non-mutated patients N = 41	p
Median age at diagnosis, years [min–max]		54.8 [33–87]	55.9 [43–78]	54.11 [33–87]	0.27
Histological subtype IDC		55 (100%)	14 (100%)	41 (100%)	1
Lymph node status	Positive	53 (96.4%)	13 (92.9%)	40 (97.6%)	1
	Negative	2 (3.6%)	1 (7.1%)	1 (2.4%)	
Molecular subtype	HER2 + and HR + /−	16 (29%)	2 (14%)	14 (34%)	0.26
	HER2− and HR−	16 (29%)	6 (43%)	10 (24%)	
	HER2− and HR +	23 (42%)	6 (43%)	17 (42%)	
Tumor grade	1–2	20 (36.4%)	6 (42.9%)	14 (34.1%)	0.79
	3	34 (61.8%)	7 (50%)	27 (65.9%)	
	NA	1 (1.8%)	1 (7.1%)	0 (0%)	
Median BMI at diagnosis kg/m^2^ [min–max]		30.6 [19–44.2]	28.6 [22.7–37.5]	31.1 [19–44.2]	0.37
Menopausal status	Premenopausal	24 (43.6%)	5 (35.7%)	19 (46.3%)	0.7
	Postmenopausal	31 (56.4%)	9 (64.3%)	22 (53.7%)	
Neoadjuvant chemotherapy		55 (100%)	14 (100%)	41 (100%)	1

*HER2 +*  HER2 positive, defined as 3 + overexpression by immunohistochemical testing or 2 + with HER2 amplification by fluorescent in-situ hybridization, *HR* hormone receptor, *BMI* body mass index, *NA * non available, *IDC* invasive ductal carcinoma.

### *PIK3CA* mutational status in tumour and corresponding plasma samples at diagnosis

**Figure 2:**
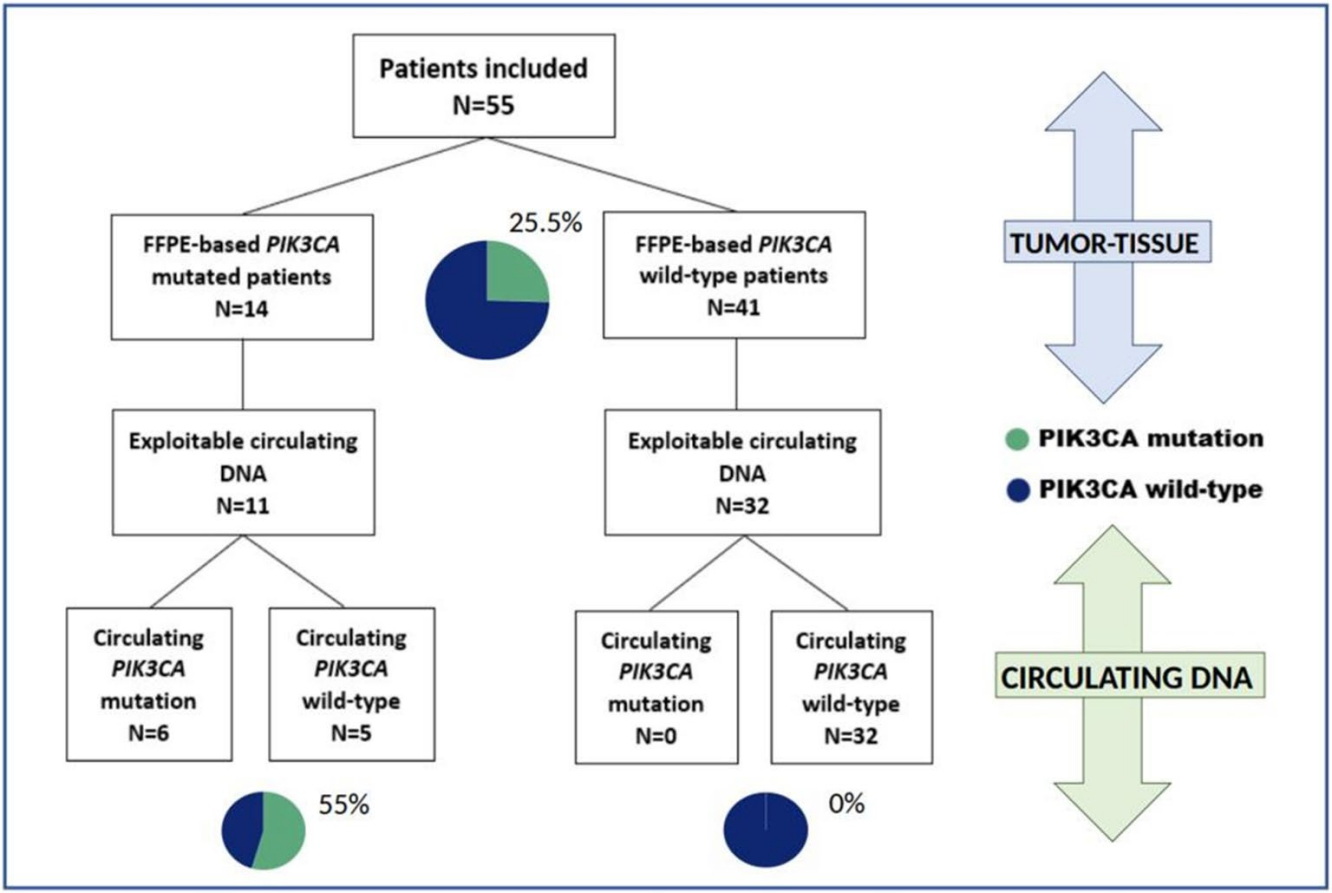
Distribution of FFPE-based and circulating *PIK3CA* mutation at diagnosis. Among the 55 patients of this cohort, FFPE-based *PIK3CA* mutation were detected in 14 patients (25.5%); among them, 11 had exploitable circulating DNA, and 6 patients (55%) harboured a corresponding circulating *PIK3CA* mutation. No other circulating mutation was identified among the 43 patients with fully interpretable circulating and biopsy mutational analyses.

A total of 14/55 patients (25.5%) had a *PIK3CA* mutation identified on baseline biopsy (H1047R = 8; H1047L = 3; E545K = 2; E542K = 1), with no significant difference in baseline characteristics between the patients with and without mutations. All mutations were single, and the prevalence of FFPE-based *PIK3CA* mutations at diagnosis was 12.5% (2/16), 26% (6/23) and 37.5% (6/16) in the HER2-positive, HR-positive/HER2-negative and HR-negative/HER2-negative subtypes, respectively. Among the 43 patients with analysable plasma samples, 6 (14%) had detectable circulating *PIK3CA* mutations, corresponding to 6/11 patients (55%) with *PIK3CA*-mutated tumours and with detectable cfDNA and 0/32 non-mutated tumours. All mutations were single. Thus, there was no additional *PIK3CA* mutation identified in ctDNA compared to FFPE-based mutational status. Those results are summarized in Fig. 2.

### Association between *PI3KCA* mutational status and pCR

A total of 52/55 patients underwent surgery; the 3 remaining patients were refractory to neoadjuvant chemotherapy. Pathological assessment in the operated patients showed 13 pCRs, 38 partial responses and 1 non-responder.

There was no difference in the pathological response rate according to *PI3KCA* tumour mutation status, with pCRs of 21.4% and 24.4% in the patients with and without mutations, respectively. Among the 14 patients with *PIK3CA* tumour mutations at diagnosis, 3/14 (21.4%) achieved a pCR after neoadjuvant chemotherapy, and 2/14 (14.3%) were non-responders (Table 2). Seven tumours had enough tissue to analyse *PIK3CA* mutation status after neoadjuvant chemotherapy. We found the same *PIK3CA* mutations as those described at diagnosis in 5 patients (71%), with a lower rate of VAF. Of note, plasma samples were available after neoadjuvant treatment in 5/14 patients with mutations (36%), with only one circulating mutation found (20%). These results are detailed in Table 2.

**Table 2:** Clinical outcomes and survival in FFPE *PIK3CA* mutated patients.

Patients	FFPE *PIK3CA* mutation at diagnosis (VAF%)	Circulating *PIK3CA* mutation at diagnosis (VAF%)	Outcomes post neoadjuvant chemotherapy	*PIK3CA* mutation on mastectomy post neoadjuvant chemotherapy (VAF%)	Circulating *PIK3CA* mutation post neoadjuvant chemotherapy (VAF%)	Relapse	DFS or follow-up (months)
No. 1	H1047L 40.3%	H1047L 11%	Pathological partial response	NA	NA	Yes	30.2
No. 2	E545K 47.80%	E545K 3.99%	Refractory	0	0	Yes	8.5
No. 3	H1047R 6.55%	NC	Refractory	NA	0	Yes	5.7
No. 4	E542K 23%	NC	pCR		NA	No	83.9
No. 5	H1047R 58.9%	H1047R 0.32%	Pathological partial response	NA	NA	No	37.6
No. 6	E545K 16.31%	E545K 6.26%	Pathological partial response	E545K 0.23%	0	Yes	8.7
No. 7	H1047R 25.3%	0	Pathological partial response	0	0	No	69
No. 8	H1047L 39.6%	H1047L 11%	Pathological partial response	H1047L 27%	H1047L 0.98%	No	20.7
No. 9	H1047R 40.7%	0	Pathological partial response	H1047R 14%	NA	No	60.4
No. 10	H1047R 41.7%	H1047R 8.13%	Pathological partial response	H1047R 41%	NA	No	9
No. 11	H1047R 3.31%	0	pCR		NA	No	71.6
No. 12	H1047R 25.2%	0	Pathological partial response	H1047R 18%	NA	No	61.9
No. 13	H1047L 0.60%	0	Pathological partial response	NA	NA	No	97.2
No. 14	H1047R 2.02%	NC	pCR		NA	Yes	47.6

*NC* non contributive, *NA* non available, *pCR* pathological complete response, *VAF* variant allele fraction.

Among the 14 patients harbouring somatic *PIK3CA* mutations, compared to 3/8 (37.5%) without circulating mutations, 0/6 patients with circulating *PIK3CA* mutations at diagnosis had pCR (p = 0.09). Thus, there is no predictive value of circulating *PIK3CA* mutations for pCR.

### Association between cfDNA and pCR

The median cfDNA level at diagnosis was 1.22 ng/µL. The rate of pCR was not different among patients above or below the median cfDNA level at diagnosis (22.2% and 25%, respectively). In contrast, 3 out of the 4 patients refractory to neoadjuvant chemotherapy had a cfDNA above 1.22 ng/µL at diagnosis. Overall, when using the median cfDNA value as the cut-off, the baseline cfDNA level was not associated with the response to neoadjuvant chemotherapy (p = 0.68). These results are detailed in Table 3.

**Table 3:** Response to neoadjuvant chemotherapy according to *PIK3CA* mutation status and cfDNA rate.

		Total N = 55 (%)	Patients without *PIK3CA* mutation N = 41 (%)	Patients with biopsy-based *PIK3CA* mutation but no corresponding circulating mutation N = 8 (%)	Patients with biopsy-based and corresponding circulating *PIK3CA* mutation N = 6 (%)	p	Patients with cfDNA ≤ 1.22 ng/µl N = 28	Patients with cfDNA > 1.22 ng/µl N = 27	p
Outcomes post neoadjuvant treatment	Pathological complete response	13 (23.6%)	10 (24.4%)	3 (37.5%)	0 (0%)	0.26	7 (25%)	6 (22.2%)	0.68
	Refractory	4 (7.3%)	2 (4.9%)	1 (12.5%)	1 (16.7%)		1 (3.6%)	3 (11.1%)	
	Pathological partial response	38 (69%)	29 (70.7%)	4 (50%)	5 (83.3%)		20 (71.4%)	18 (66.7%)	

*cfDNA* cell-free DNA.

### Association between *PIK3CA* mutation status and cfDNA with OS and DFS

After a median follow-up of 52.1 months [7.7–140.6], the median OS was not reached in our retrospective cohort, with 20 deaths among our 55 included patients. No significant difference was found in OS according to FFPE-based *PIK3CA* mutation status at diagnosis (HR = 0.95 CI[0.35–2.63], p = 0.93), according to circulating *PIK3CA* mutation status at diagnosis (HR = 2.27 CI[0.66–7.81], p = 0.18) and according to circulating and biopsy-based *PIK3CA* mutation status at diagnosis (p = 0.29).

The median DFS was 104.8 months, and 22 relapses were observed. No significant difference was found in DFS according to FFPE-based *PIK3CA* mutation status at diagnosis (HR = 0.92 CI[0.34–2.52], p = 0.88), according to circulating *PIK3CA* mutation status at diagnosis (HR = 2.24 CI[0.64–7.81], p = 0.19) and according to circulating and biopsy-based *PIK3CA* mutation status at diagnosis (p = 0.29).

**Figure 3:**
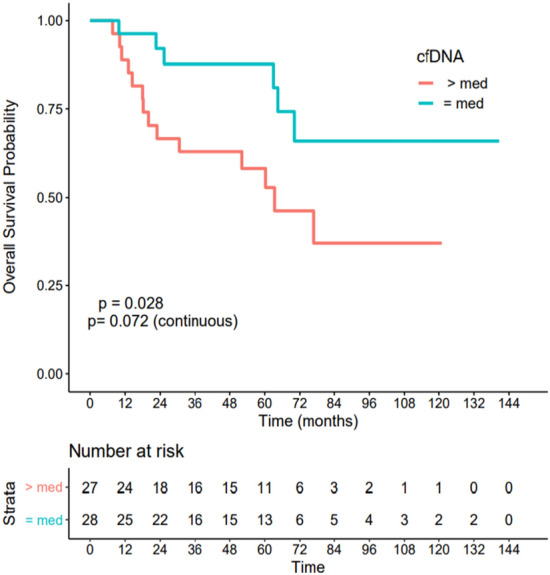
Association between cell-free DNA level at diagnosis and overall survival. Patients with cfDNA below the median had a significantly better OS outcome (HR = 0.36 CI[0.14–0.93], p = 0.028).

**Figure 4:**
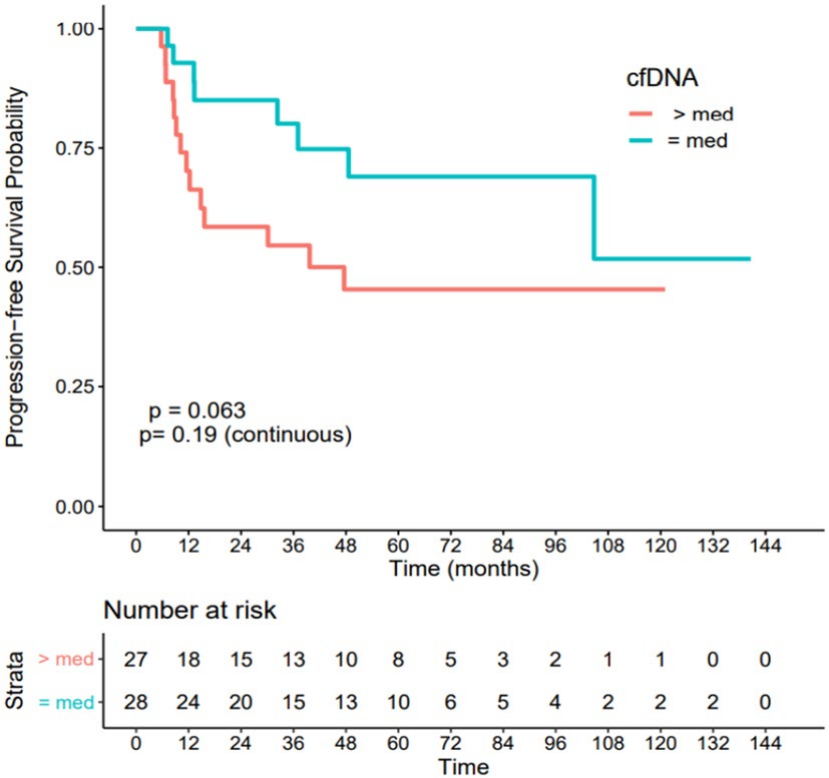
Association between cell-free DNA level at diagnosis and disease-free survival. Patients with cfDNA below the median had a non-significant better DFS outcome (HR = 0.45 CI[0.19–1.07], p = 0.063).

Using the median baseline cfDNA level as the threshold, the patients with low cfDNA had a significantly better OS outcome (HR = 0.36 CI[0.14–0.93], p = 0.028) Fig. 3. Regarding DFS, a non-significant trend also identified a low baseline cfDNA level at diagnosis as a marker of better outcome (HR = 0.45 CI[0.19–1.07], p = 0.063), Fig. 4.

Indeed, the 3-year DFS rate was 54.6% [38.5–77.4] for patients with cfDNA greater than the median value of 1.22 ng/µL and 80.1% [65.7–97.7] for patients with cfDNA equal to or lower than 1.22 ng/µL. Similarly, the 3-year OS rates were 63% [47.1–84.1%] and 87.7% [75.6–100], respectively.

### Association between pCR and survival data

**Figure 5:**
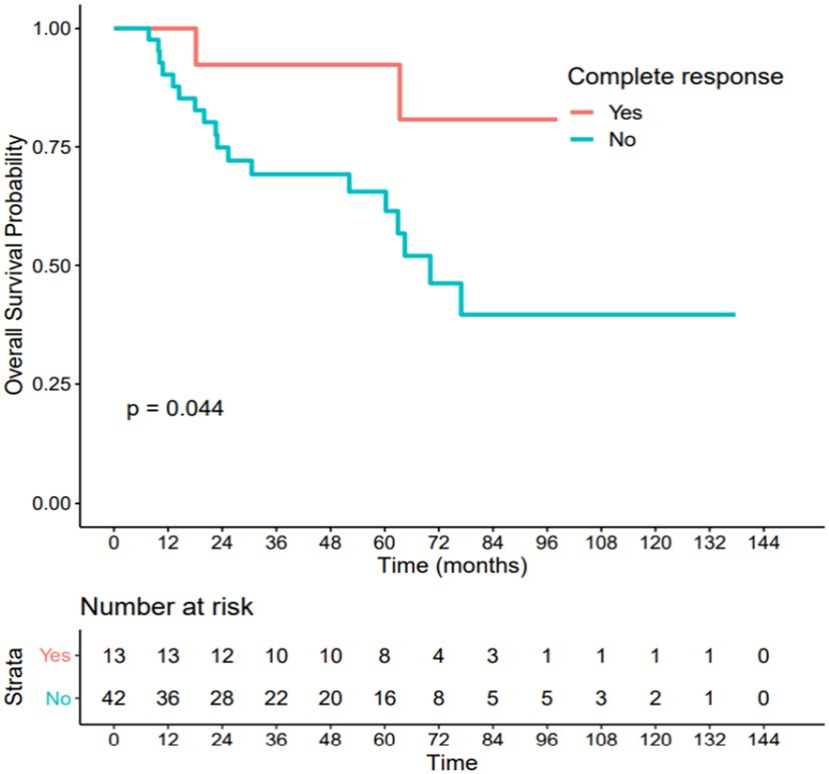
Association between pCR and overall survival. Patients with pCR had a significantly better overall survival (HR = 0.25 IC[0.06–1.08], p = 0.044).

**Figure 6:**
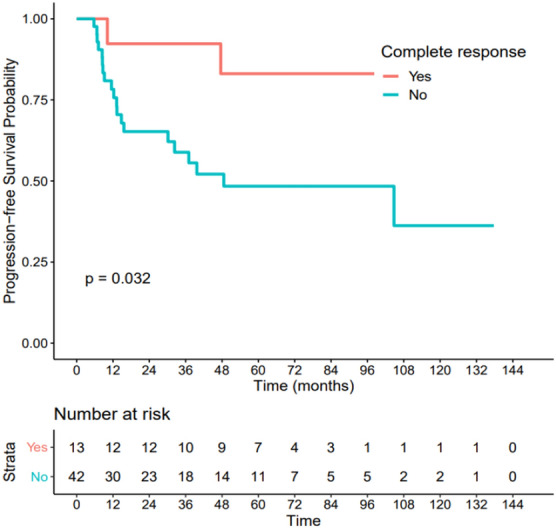
Association between pCR and disease-free survival. Patients with pCR had a significantly better disease-free survival (HR = 0.23 IC[0.05–1], p = 0.032).

Among the 13 patients with pCR after neoadjuvant treatment, only 2 (15%) experienced tumoral relapse during follow-up, compared to 20 relapses among the 42 patients with partial or refractory histological response (47.6%). A significant difference was found in OS according to the response to neoadjuvant treatment (HR = 0.25 IC[0.06–1.08], p = 0.044) Fig. 5, and in DFS (HR = 0.23 IC[0.05–1], p = 0.032), Fig. 6.

## Discussion

This retrospective study included 55 LAIBC patients, among which 25.5% had *PIK3CA*-mutated tumours. Corresponding circulating *PIK3CA* mutations were identified in 55% of patients with mutations, while no circulating mutations were found among patients with *PI3KCA* WT tumours. There was no predictive value for pCR of *PIK3CA* mutations or baseline cfDNA level and no prognostic value of *PIK3CA* mutation status. In contrast, patients with baseline cfDNA below the median or those with pCR after NCT had a better prognosis.

To our knowledge, this study is the first to address circulating *PIK3CA* mutations in patients treated for LAIBC.

Indeed, IBC is a sub-type excluded from most studies, including SOLAR-1^29^. We found one study dealing with cell-free DNA in 19 patients with IBC, but only one *PIK3CA* mutation was found in tumour samples without circulating corresponding mutations^32^.

Despite the limited number of patients included, our cohort seems representative of the non-metastatic IBC population. Indeed, this cohort was characterized by a majority of obese patients, with an aggressive tumoral profile, as already described in the IBC population^33,34^. As expected, a higher rate of HER2-positive and triple-negative tumours in comparison to non-inflammatory breast cancer was observed, as well as a pCR rate of 23.6%, comparable to the pCR rate of 23.2% reported in the study of Van Uden et al.^11^. In our study, a *PIK3CA* mutation was found at a rate of 25.5% on initial biopsy, corroborating recently published data about IBC, with a rate of 29.5% among 156 patients and a rate of 28% among 53 patients, reported by Liang et al. and Ross et al., respectively^35,36^. Similar results were described in non-inflammatory early-stage breast cancer, with a rate of 32% among 10 319 patients and a rate of 23% among 1008 patients, reported by Zardavas et al. and Papaxoinis et al.^37,38^. Only single-hotspot mutations were detected in this study, whereas multiple *PIK3CA* mutations were described in 12 to 15% of *PIK3CA*-mutated breast cancers^39–41^. As expected, a majority of *PIK3CA* mutations were localized in the H1047R hotspot in exon 20^42,43^. Our results highlight that a corresponding circulating *PIK3CA* mutation was identified in 55% of non-metastatic IBC patients with a baseline somatic *PIK3CA* mutation in tumour tissue and with detectable cfDNA, while no circulating mutation was found among patients with no *PIK3CA* mutations. Despite its aggressiveness, *PIK3CA*-mutated LAIBC appears to have a *PIK3CA* circulating detection rate comparable to localized (47%) rather than metastatic breast cancer (approximately 80%)^23,44,45^. Thus, those results do not encourage the use of cfDNA testing to find actionable findings earlier during patient management. Based on the favourable results of the SOLAR-1 study, therapeutic trials are expected in *PIK3CA*-mutated positive hormone receptor LAIBC with the use of alpelisib in neoadjuvant treatment or in therapeutic intensification after surgery with residual invasive cancer. In our study, *PIK3CA* mutation status does not appear to have prognostic value, as in non-inflammatory early breast cancer, or predictive value, but no definite conclusion can be formulated given the small number of patients with mutations.

Interestingly, our results highlight the prognostic value of baseline cfDNA, showing worse survival outcome for LAIBC patients with cfDNA above the median, suggesting that baseline cfDNA could reflect tumour burden in LAIBC. The predictive and prognostic value of cfDNA has been demonstrated in several studies, mostly in lung cancer^46^, rectal cancer during neoadjuvant chemotherapy^47^, and metastatic breast cancer^30^. In the study of Park et al., among 72 early-stage triple-negative breast cancer patients who underwent NCT, patients with baseline cfDNA levels > 264 ng/mL demonstrated a higher risk of relapse than those with baseline cfDNA levels ≤ 264 ng/mL (HR, 2.84; 95% CI, 1.11–7.24; P = 0.029)^48^. Otherwise, as expected, pathological complete response (pCR) after neoadjuvant treatment in LAIBC is a predictor of favourable long-term outcome, corroborating literature data. Indeed, among 1061 early breast cancer patients of all subtypes, improved survival was previously reported for patients who achieved pCR, especially for HER2 + /HR− tumour subtypes with a 5-year overall survival rate of 83% with pCR versus 50% without pCR^49^. Similarly, Pierga et al. demonstrated the prognostic value of pCR and circulating tumour cells rate at baseline in inflammatory breast cancer in a pooled analysis of BEVERLY-1 and -2^50^.

Our study has some limitations. First, given the limited number of patients, our results cannot be considered definitive. Nevertheless, it must be taken into consideration that IBC is a rare disease, explaining the limited literature data available. Moreover, the confirmation of the pCR status and cfDNA level as prognostic factors highlights the internal validity of our results. Second, due to its retrospective design, some FFPE or plasma samples could not be used, with a lack of quality DNA mostly due to storage constraints and long storage times. Moreover, taking into account a limited quantity of material and a majority of heparinized plasma samples, we could not study genomic alterations by targeted next-generation sequencing. Taken together, these technical limitations prevented us from studying genomic tumoral heterogeneity which could have provided precious new information within the mutational landscape of IBC.

Finally, since we focused our analysis on the four main *PIK3CA* mutations by ddPCR, we cannot exclude the presence of rare mutations, and we could not analyse *AKT* mutations or *PTEN* deletion that result in the same oncogenic activation pathway, which could participate in the resistance mechanisms of *PIK3CA* therapies.

## Conclusion

In conclusion, this study showed that a corresponding circulating *PIK3CA* mutation was identified in 55% of non-metastatic IBC patients with baseline somatic *PIK3CA* mutations in tumour tissue and with detectable cfDNA, while no circulating mutation was found among patients with no *PIK3CA* mutations. Despite its aggressiveness, LAIBC surprisingly appears to have quite a low circulating ctDNA release. These results suggest that future therapeutic trials based on *PIK3CA* mutation status within LAIBC should focus mostly on primary material. Nevertheless, the cfDNA rate seems to be a discriminatory predictor of survival, allowing us to better stratify patients according to their level of risk (Suppl. Information).

## Supplementary Information

Supplementary Information.

## Data Availability

The datasets generated during and/or analysed during the current study are available from the corresponding author on reasonable request.

## Abbreviations

AJCC: American Joint Committee of Cancer
BC: Breast cancer
BMI: Body mass index
CI: Confidence interval
ctDNA: Circulating tumoral DNA
cfDNA: Cell-free DNA
ddPCR: Digital droplet polymerase chain reaction
DFS: Disease-free survival
FFPE: Formalin-fixed, paraffin-embedded
HR: Hormone receptor
IBC: Inflammatory breast cancer
IRB: Institutional Review Board
LAIBC: Locally-advanced inflammatory breast cancer
LOD: Limit of detection
NCT: Neoadjuvant chemotherapy
OS: Overall survival
pCR: Pathological complete response
VAF: Variant allele fraction
WT: Wild-type
