# Supplementary material for: Circulating PIK3CA mutation detection at diagnosis in non-metastatic inflammatory breast cancer patients
Source: Sci Rep. 2021 Dec 15;11:24041. doi: 10.1038/s41598-021-02643-y (PMC8674263; doi:10.1038/s41598-021-02643-y)
Supplement: Supplementary file 1 — Supplementary Information. [file 41598_2021_2643_MOESM1_ESM.docx]

**SUPPLEMENTARY DATA**

**METHODS**

**Pre-analytical conditions**

Tissue blocks and plasma samples were stored during 1 to 11 years according to the date of the start of care for each patient included. This large delay may be implicated in a decrease of ctDNA in samples (1), probably more than the temperature storage for plasmas (2). Moreover, most of our plasma samples were heparinized plasmas, with a known risk of PCR inhibition. All these pre-analytical conditions may induce a lack of detection of ctDNA.

We focused on several points to optimize our assays:

- At collection time, whole blood samples were quickly processed, with a first centrifugation at 2000g (10min) within 2 hours after collection, to reduce the potential contamination of plasma by white blood cells (3)
- Stored at -20°C, the plasma samples were not thawed before DNA extraction for our study
- We performed a pre-amplification before ddPCR, to increase the total copy number of DNA in our samples, as previously reported (4,5)
- For DNA extracted from heparinized plasmas, we used *Bacteroides* Heparinase I (New England Biolabs, USA) during the pre-amplifcation step to limit deleterious impact of heparin (6) on PCR amplification

Using a pre-amplification of DNA and Heparinase I enables us to explore 43 of the 55 collected plasmas: for 12 plasmas, less than 200 copies/µL were obtained with ddPCR, questioning the extracted DNA quality, and thus the analysis relevance.

**ddPCR**

1. General information on ddPCR:

In ddPCR, DNA isolated from biological samples is partitioned into multiple compartments (about 30 000 individual droplets generated by water-in-oil emulsion, in the Naica™ System (Stilla Technologies, Villejuif, France)). In each compartment, separate real-time PCR reactions are performed using fluorescent probes that specifically target mutant or wild-type DNA copies (7). Based on Poisson statistics, either one or zero DNA molecule is initially present within a partition: thus, all amplicons generated in each droplet derive from a single DNA molecule. Through a digital counting of mutant or wild-type fluorescent signals, an absolute quantification of mutant DNA copies and wild-type DNA copies is performed, with no need to rely on standard calibration curves.

1. *PIK3CA* mutation detection and data analysis

Analyses for *PIK3CA* mutation detection were performed blind to clinical data. ddPCR from the Stilla system (Stilla Technologies, Villejuif, France) was used for *PIK3CA* mutation detection in the plasma and FFPE samples. We used a Bio-Rad (Hercules, CA, USA) ddPCR assay for the four mutations, E542K (dHsaMDV2010073), E545K (dHsaMDV2010123), H1047R (dHsaMDV2010077), and H1047L (dHsaMDV2010123). Bio-Rad ddPCR assay combine free nucleotides, primers, and 2 probes: one probe specific of the wild type sequence, combined with the HEX fluorophore, and another probe, specific of the mutant sequence, combined with the FAM fluorophore. To analyze the 4 hot spots for *PIK3CA* mutations (E542K, E545K, H1047R/L), we performed 4 independent ddPCR, with 4 different Bio-Rad ddPCR assays, designed for each mutation. After the ddPCR run, every wild type or mutant *PIK3CA* DNA copy was detected, according to the fluorescent emission, and counted. ddPCR enables an absolute quantification of mutant or wilt-type *PIK3CA* DNA copies. These assays are designed by Biorad following the MIQE guideline for commercial assay recommendation.

We validated the specificity of our assays using known positive controls: NGS characterized cell lines (NCI-H460 for E545K mutation, T84 for E542K mutation), commercial control samples (Quality Control HD701, Horizon Discovery, Cambridge, UK) for H1047R), or tumor sample with H1047L mutation, controlled by Sanger sequencing.

*PIK3CA* pseudogene has been described on chromosome 22, spanning exons 9 through 13 with >95% sequence homology (8). As H1047R and H 1047L mutations are in exon 20, these assays are not concerned with the pseudogene. On the contrary, regarding E545K and E542K mutations, the assays might be biased by PIK3CA pseudogene (9). For codon 545, wild type gene and pseudogene differs on 1 nucleotide: GAG for wild type sequence, and GCG for pseudogene; in case of mutation on codon 545, the resulting sequence is AAG. For codon 542, wild type gene and pseudogene have the same sequence, GAA; in case of mutation on codon 542, the resulting sequence is AAA.

Anyway, we rely on Biorad’s expertise to avoid a *PIK3CA* pseudogene interference: *PIK3CA* Biorad assays are used in ddPCR by several teams, in the context of breast cancer (10) or not (11).

Finally, we cannot definitively exclude a possible interference with *PIK3CA* pseudogene.

The variant allele fraction (VAF) was defined as the proportion of mutant DNA copies compared with wild-type (WT) DNA copies obtained by ddPCR.

To validate the run, we verified 2 criteria: a minimum of 15000 total droplets generated, and a minimum of 200copies/µL (wild type copies + mutant copies) obtained. In contributive runs according to these 2 previous criteria, we confronted the number of mutant positive droplets to the limit of detection (LOD) value: the sample was considered as positive if the number of positive droplets was larger than the LOD. Each sample was tested in duplicated. For every duplicate, the same qualitative conclusion (mutated or not mutated) was obtained. The VAF mentioned in table 2 of the article represents the mean of the 2 duplicates.

The results were analyzed using CrystalMiner software (Stilla Technologies, Villejuif, France), which enables a visualization of each chamber (visualization of the droplets, appearing as empty, wild-type positive or mutant positive) and provides a count of the generated droplets, the positive droplets for wild-type signal and the positive droplets for mutant signal.

LOD have been determined according to the instructions of “Additional support information for digital PCR statistics on the Naica Platform (LOB, LOD, CNV), Version:1.2” (Stilla Technologies, Villejuif, France), using healthy control samples (5 to 8 healthy control samples tested for each mutation and each type of mutations). Our approach was similar as in Milbury et al. (12) or Corné et al. (13)

For each type of material (DNA extracted from FFPE, heparinized or EDTA samples), we determined the LOB (Limit of Blank) and LOD (Limit of Detection). To perform our analysis of the ddPCR runs, we expressed these LOB and LOD in number of positive droplets, for each *PIK3CA* mutations: we consider a sample as “mutated” if the number of mutant positive droplets was superior to the LOD.

The LOB and the LOD can also be expressed in copies per µL.

**Table 1: LOD of the 4 *PIK3CA* mutations**

| mutation | H1047L | | | H1047R | | | E545K | | | HE542K | | |
| --- | --- | --- | --- | --- | --- | --- | --- | --- | --- | --- | --- | --- |
| samples | FFPE | Heparin  plasma | EDTA plasma | FFPE | Heparin plasma | EDTA plasma | FFPE | Heparin  plasma | EDTA plasma | FFPE | Heparin plasma | EDTA plasma |
| LOB (95%) in number of positive droplets | 6 | 2 | 5 | 5 | 4 | 4 | 8 | 3 | 4 | 10 | 2 | 6 |
| LOD (95%) in number of positive droplets | 12 | 7 | 11 | 11 | 9 | 9 | 15 | 8 | 9 | 17 | 7 | 12 |
| LOD (95%) in cp/µL | 1.56 | 0.8 | 1.01 | 1.23 | 1.24 | 0.91 | 1.36 | 0.95 | 0.81 | 1.61 | 0.65 | 1.2 |

To test the sentivity of the 4 assays, DNA mixes were prepared using serial dilution DNA from mutated samples in WT DNA. The DNA mixes were assayed in triplicate, and we confronted the number of mutant positive droplets to the LOD value: the sample was considered as positive if the number of positive droplets was larger than the LOD. We obtained sensitivities below 0.2% for the 4 assays.

1. Sozzi G, Roz L, Conte D, Mariani L, Andriani F, Verderio P, et al. Effects of prolonged storage of whole plasma or isolated plasma DNA on the results of circulating DNA quantification assays. J Natl Cancer Inst. 21 déc 2005;97(24):1848‑50.

2. El Messaoudi S, Rolet F, Mouliere F, Thierry AR. Circulating cell free DNA: Preanalytical considerations. Clin Chim Acta Int J Clin Chem. 23 sept 2013;424:222‑30.

3. Geeurickx E, Hendrix A. Targets, pitfalls and reference materials for liquid biopsy tests in cancer diagnostics. Mol Aspects Med. avr 2020;72:100828.

4. Clatot F, Perdrix A, Augusto L, Beaussire L, Delacour J, Calbrix C, et al. Kinetics, prognostic and predictive values of ESR1 circulating mutations in metastatic breast cancer patients progressing on aromatase inhibitor. Oncotarget. 15 2016;7(46):74448‑59.

5. Allouchery V, Beaussire L, Perdrix A, Sefrioui D, Augusto L, Guillemet C, et al. Circulating ESR1 mutations at the end of aromatase inhibitor adjuvant treatment and after relapse in breast cancer patients. Breast Cancer Res. 16 mai 2018;20(1):40.

6. Sefrioui D, Beaussire L, Clatot F, Delacour J, Perdrix A, Frebourg T, et al. Heparinase enables reliable quantification of circulating tumor DNA from heparinized plasma samples by droplet digital PCR. Clin Chim Acta Int J Clin Chem. sept 2017;472:75‑9.

7. Perkins G, Lu H, Garlan F, Taly V. Droplet-Based Digital PCR: Application in Cancer Research. Adv Clin Chem. 2017;79:43‑91.

8. Baker CL, Vaughn CP, Samowitz WS. A PIK3CA pyrosequencing-based assay that excludes pseudogene interference. J Mol Diagn JMD. janv 2012;14(1):56‑60.

9. Han N, Cheng Q-Y, Chen B, Cai J-F, Wang X-J, Lou C-J, et al. PIK3CA Mutations in Resected Small Cell Lung Cancer. Adv Clin Exp Med Off Organ Wroclaw Med Univ. juin 2016;25(3):397‑402.

10. Moynahan ME, Chen D, He W, Sung P, Samoila A, You D, et al. Correlation between PIK3CA mutations in cell-free DNA and everolimus efficacy in HR + , HER2 − advanced breast cancer: results from BOLERO-2. Br J Cancer. mars 2017;116(6):726‑30.

11. Kim ST, Lira M, Deng S, Lee S, Park YS, Lim HY, et al. PIK3CA mutation detection in metastatic biliary cancer using cell-free DNA. Oncotarget. 24 nov 2015;6(37):40026‑35.

12. Milbury CA, Zhong Q, Lin J, Williams M, Olson J, Link DR, et al. Determining lower limits of detection of digital PCR assays for cancer-related gene mutations. Biomol Detect Quantif. sept 2014;1(1):8‑22.

13. Corné J, Le Du F, Quillien V, Godey F, Robert L, Bourien H, et al. Development of multiplex digital PCR assays for the detection of PIK3CA mutations in the plasma of metastatic breast cancer patients. Sci Rep. 27 août 2021;11(1):17316.
